# Microglial CARD19 ameliorates post-stroke neuroinflammation by stabilizing mitochondrial cristae

**DOI:** 10.4103/NRR.NRR-D-24-00923

**Published:** 2025-03-25

**Authors:** Yujie Hu, Liwen Zhu, Chao Zhou, Qi Li, Huiya Li, Shiji Deng, Shengnan Xia, Haiyan Yang, Xinyu Bao, Pinyi Liu, Yun Xu

**Affiliations:** 1 Department of Neurology, Nanjing Drum Tower Hospital, Affiliated Hospital of Medical School, Nanjing University, Nanjing, Jiangsu Province, China; 2 Department of Neurology, Nanjing Drum Tower Hospital, State Key Laboratory of Pharmaceutical Biotechnology and Institute of Translational Medicine for Brain Critical Diseases, Nanjing University, Nanjing, Jiangsu Province, China; 3 Department of Neurology, Nanjing Drum Tower Hospital, Clinical College of Traditional Chinese and Western Medicine, Nanjing University of Chinese Medicine, Nanjing, Jiangsu Province, China; 4 Jiangsu Provincial Key Discipline of Neurology, Nanjing, Jiangsu Province, China; 5 Jiangsu Key Laboratory for Molecular Medicine, Medical School of Nanjing University, Nanjing, Jiangsu Province, China

**Keywords:** apoptosis, CARD19, ischemic stroke, microglia, mitochondrial cristae, mitochondrial DNA, mitochondrial dysfunction, mitochondrial intermembrane bridge, mitochondrion, neuroinflammation

## Abstract

Microglia are the first immune cells that are activated in the brain following ischemic stroke. Mitochondrial dysfunction exacerbates microglia-mediated neuroinflammation post-stroke. Caspase activation and recruitment domain 19 (CARD19) is involved in innate immune response and inflammatory response, which are also important functions of microglia. However, the role of CARD19 in microglial biology and ischemic stroke remains unknown. Here, we observed that CARD19 expression was significantly elevated in microglia in the penumbra after ischemic stroke via analyzing the spatial transcriptomic sequencing data of ischemic brain tissue, as well as in an *in vitro* model of microglial activation. Remarkably, conditional knockdown of *Card19* in microglia promoted post-stroke neuroinflammation and worsened neurological outcomes in a mouse model of ischemic stroke. Mechanistically, we found that CARD19 localized to mitochondria and promoted the assembly of mitochondrial intermembrane bridge components, while CARD19 deficiency in microglia caused ultrastructural and functional damage to the mitochondrial cristae, leading to an exaggerated pro-inflammatory response. Thus, our findings suggest that preserving mitochondrial cristae, by targeting CARD19 could be a novel therapeutic strategy for ameliorating neuroinflammation post-stroke and decreasing the volume of the ischemic penumbra.

## Graphical abstract

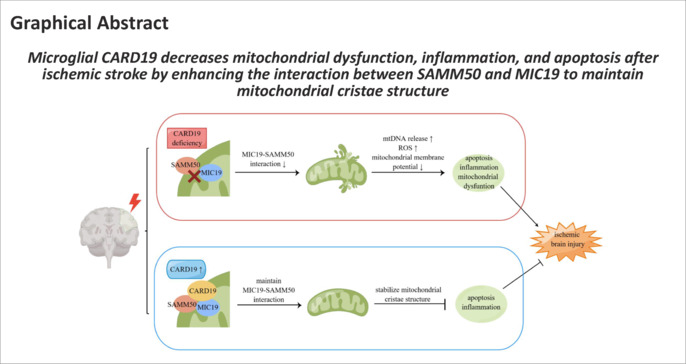

## Introduction

Ischemic stroke is a common cause of death and disability worldwide (Lambertsen et al., 2019). Microglia are the resident immune cells in the central nervous system and play a key role in immune surveillance and maintenance of brain homeostasis (Qin et al., 2019; Zheng et al., 2025). After ischemic stroke, dying neurons release a large number of damage-associated molecular patterns (DAMPs) that activate microglia, which then perform multiple immune functions, including phagocytosis, cytokine secretion, and reactive oxygen species (ROS) generation (Ransohoff and Perry, 2009). Specifically, during the acute phase of ischemic stroke, activated microglia release numerous pro-inflammatory factors and chemokines, such as interleukin (IL)-1β, IL-6, and tumor necrosis factor-α (TNF-α), which further damage neurons, destroy the blood–brain barrier, and recruit peripheral immune cells to infiltrate the ischemic area, aggravating brain injury (Jayaraj et al., 2019).

Mitochondria, traditionally viewed as the cellular powerhouses, have important functions beyond energy production (Monzel et al., 2023; Peng et al., 2024; Qin et al., 2025; Xu et al., 2025). Accumulating evidence has highlighted the role of mitochondria in the immune response (Mills et al., 2017; Marchi et al., 2023). Damaged mitochondria release components including cardiolipin, circular mitochondrial DNA (mtDNA), mitochondrial ROS, mitochondrial RNA, and N-formylmethionine (the amino acid encoded by the start codon), into the cytoplasm. These components act as DAMPs, initiating cellular immune responses (Shen et al., 2022). Limiting mtDNA synthesis and oxidized mtDNA formation inhibits NOD-like receptor family pyrin domain containing 3 (NLRP3) inflammasome activation in microglia, thereby alleviating neuroinflammation after ischemic stroke (Guan et al., 2024). Moreover, mitochondrial energy metabolism induces microglial polarization, thereby affecting pathological inflammatory responses (Orihuela et al., 2016; Bernier et al., 2020; Chen et al., 2023). In response to inflammatory stress, microglial metabolism shifts from oxidative phosphorylation to glycolysis to offset energy deficits (Preeti et al., 2022). Increased glycolytic flux regulates the immune functions of microglia, such as cytokine production and phagocytosis, thus promoting neuroinflammation in the brain (Bernier et al., 2020). In addition, mitochondrial complex I sustains microglial activation by facilitating reverse electron transport and ROS production (Peruzzotti-Jametti et al., 2024).

CARD19 belongs to the caspase recruitment domain family (Rios et al., 2020). A previous study reported that CARD19 localizes to mitochondria (Chen et al., 2013). CARD19 inhibits BCL10-induced NF-κB activation and promotes BCL10 degradation through CARD–CARD domain interactions (Woo et al., 2004; Rios et al., 2020). CARD19 also negatively regulates BCR/TAK1-induced NF-κB activation in B cells (Zheng et al., 2023). A recent study showed that CARD19 interacts with mitochondrial contact site and cristae organizing system (MICOS) to regulate cristae morphology, suggesting that CARD19 may be a potential component or regulator of the mitochondrial intermembrane bridge (MIB) (Rios et al., 2022). Loss of oxygen and energy after ischemic stroke can lead to mitochondrial damage (Sims and Muyderman, 2010). Given that CARD19 has been reported to regulate mitochondrial structure, it suggests that it may play a role after ischemic stroke.

The aim of this study was to elucidate the role of microglial CARD19 in ischemic stroke. We hypothesized that CARD19 mitigates post-stroke neuroinflammation and rescues mitochondrial dysfunction by stabilizing mitochondrial cristae structure during the acute phase of ischemic stroke. Gaining a better understanding of CARD19 function and the underlying mechanisms may lead to novel therapeutic strategies for ischemic stroke.

## Methods

### Animals

**Figure 1:**
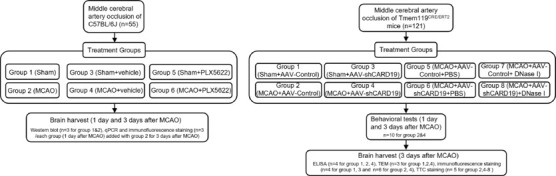
Study flowchart. AAV: Adeno-associated virus; ELISA: enzyme-linked immunosorbent assay; MCAO: middle cerebral artery occlusion; PLX5622: CSF1R inhibitor; qPCR: quantitative polymerase chain reaction; TEM: transmission electron microscopy; TTC: 2,3,5-triphenyltetrazolium chloride; WB: western blotting.

**Figure 2:**
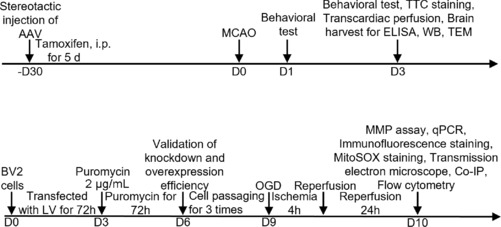
Experimental timeline. AAV: Adeno-associated virus; Co-IP: Co-immunoprecipitation; ELISA: enzyme-linked immunosorbent assay; OGD: oxygen–glucose deprivation/reoxygenation; qPCR: quantitative polymerase chain reaction; TEM: transmission electron microscopy; TTC: 2,3,5-triphenyltetrazolium chloride; WB: western blotting.

Male C57BL/6J mice (8 weeks old, 22–25 g) were purchased from GemPharmatech, Nanjing, China (license No. SCXK (Su) 2018-0008). Male and female Tmem119^Cre/ERT2^ mice were purchased from Model Organisms, Shanghai, China (license No. SCXK (Hu) 2019-0002). Tmem119 is a specific marker of microglia in the brain (Bennett et al., 2016). Hence, Tmem119^Cre/ERT2^ mice can used for the study of microglia in the brain and have been used in this capacity in several studies (Liu et al., 2023; Lan et al., 2024; Li et al., 2024b). Considering the potential impact of estrogen on experimental outcomes (Sommer, 2017), only male mice were used in this study. The experimental mice were housed under specific-pathogen–free conditions at a temperature of **~**22°C and a humidity level of 40%–70%, with a 12/12-hour light/dark cycle. The mice were provided with adequate food and water every day and housed five per cage. The anesthesia details are described below. All the animal procedures were reviewed and approved by Animal Care and Use Committee at Nanjing University (approval No. 2023AE01022) and conducted in strict accordance with international laws and National Institutes of Health policies, including the Guide for the Care and Use of Laboratory Animals (8^th^ ed., National Research Council, 2011). This study is reported in accordance with the ARRIVE 2.0 guidelines (Animal Research: Reporting of *In Vivo* Experiments) (Percie du Sert et al., 2020). The study design and animal grouping are shown in **Figures 1** and **2**.

### Middle cerebral artery occlusion model

Male C57BL/6J and Tmem119^Cre/ERT2^ mice were anesthetized with a mixture of 1.5% isoflurane and 30% oxygen (RWD, Shenzhen, China). The internal carotid artery and external carotid artery were exposed under a dissecting microscope. Then, a small incision was made in the internal carotid artery, and a 6-0 nylon suture was inserted into the artery and advanced until resistance was felt. During the operation, a doppler blood flow detector was used to monitor right cerebral blood flow. When the blood flow dropped to 70% of the initial flow, a knot was tied at the end of the suture to keep it in place. After 60 minutes of occlusion, the suture was removed. Mice were placed on a heating pad while they recovered from the anesthesia.

### Depletion of microglia

C57BL/6J mice were fed with 8 g feed containing PLX5622 (a CSF1R inhibitor) (each kilogram of feed contains 1200 mg PLX5622; SYSE BIO, Changzhou, China, Cat# 20010801) daily for 2 weeks to deplete microglia.

### Cerebral blood flow measurement

After the mice were anesthetized, an incision was made to expose the skull. An RFLSI ZW laser speckle flow imaging system (RWD) was used to record cerebral perfusion 10 minutes before middle cerebral artery occlusion (MCAO), 10 minutes after surgery, and 30 minutes after reperfusion. RFLSI Analysis software (RWD) was used to analyze regional cerebral blood flow (rCBF). The relative rCBF was the calculated as follows: ipsilateral rCBF/contralateral rCBF.

### Stereotaxic intracranial injection

Stereotaxic intracranial injection was performed as previously described (Ge et al., 2023). Briefly, the mice were anesthetized with a mixture of 1.5% isoflurane and 30% oxygen (RWD) and immobilized on an operating table. Then, the skull, bregma, and posterior fontanelle were exposed. The three targeted sites for injection were determined based on the brain atlas with the following coordinates: 3 mm to the right of the midline; 0.3 mm posterior to bregma, 0.9 mm and 1.8 mm anterior to bregma; and 1.8 mm ventral from bregma (Paxinos and Franklin, 2012). A total of 200 nL of different viruses was injected at each site at a rate of 20 nL/min. Adeno-associated virus (AAV) virus was purchased from Brainvta (Wuhan, China), and rAAV6m-CMV-DIO-(mCherryU6)-shRNA (Card19)-WPRE-hGH polyA was constructed by inserting the target sequence GAA CGG CAC TGT CAG GAA TTT. The control mice were injected with AAV-control (no functional shRNA). Stereotaxic injection was followed by intraperitoneal injection of tamoxifen (MCE, Shanghai, China, Cat# HY-13757A) at a dose of 75 mg/kg per day for 5 days to induce expression of the recombinant enzyme Cre in Tmem119^Cre/ERT2^ mice. The mice underwent MCAO surgery 25 days after stereotaxic injection.

### Injection of deoxyribonuclease I

Mice in the AAV-control and AAV-shCARD19 groups received intraperitoneal injections of deoxyribonuclease I (DNase I) (3 mg/kg per day; Roche, Basel, Switzerlan, Cat# 10104159001) for 3 consecutive days following MCAO to eliminate cytoplasmic mtDNA.

### Sensorimotor behavioral tests

Neurological impairment after MCAO was evaluated by the foot fault test, modified neurological severity score (mNSS), the rotarod test, and the grip strength test, as previously described (Ge et al., 2023). The foot fault test was performed 3 days before MCAO for training. The mice were allowed to walk freely on the stainless-steel grid twice a day for 5 minutes each time. Walking videos were taken before and after MCAO. The proportion of foot faults was calculated by dividing the number of wrong steps taken with the left forelimb by the total number of steps taken with the left forelimb. The mNSS test assesses four aspects: reflex, sensory, motor, and balance function. mNSS scores range from 0 to 12, with higher scores indicating more severe neurological deficits. The rotarod test was used to evaluate motor function, coordination, and fatigue. The mice were trained before MCAO to balance on a 40 r/min roller for 5 minutes without falling. After MCAO, the time that it took the mice to fall off the roller was recorded. The grip strength test was used to measure the maximum forelimb grip strength of the mice.

### Infarct volume measurement

2,3,5-triphenyltetrazolium chloride (TTC) staining was used to measure infarct volume. Whole mouse brains were sliced into 1-mm-thick coronal sections and soaked in 2% TTC solution (Sigma-Aldrich, Darmstadt, Germany) for 20 minutes. ImageJ (version 1.53o11, NIH, Bethesda, MD, USA) was used to calculate the infarct volume. Infarct volume ratio = (contralateral area – ipsilateral non-infarct area)/contralateral area × 100%.

### Spatial transcriptomic analysis

**Figure 3:**
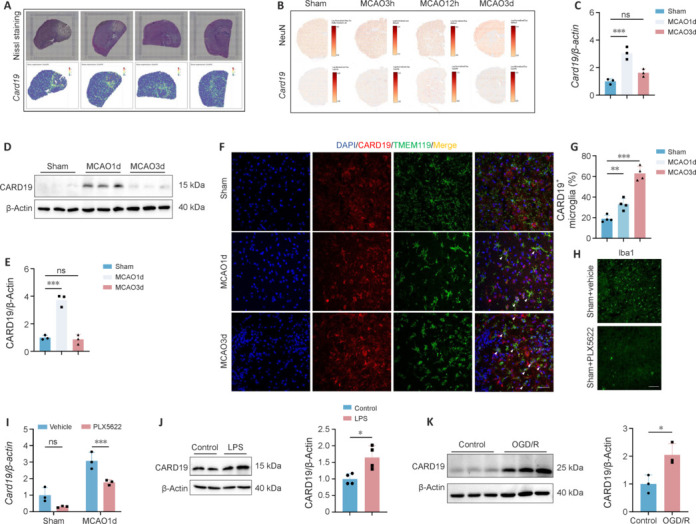
CARD19 expression is increased in microglia after ischemic stroke. (A) Spatial transcriptomic analysis of *Card19* expression 3 hours, 12 hours, and 3 days after MCAO. (B) Spatially resolved heatmaps across tissue sections showing spatial gene expression patterns in mice 3 days after photothrombosis. (C) qPCR analysis of ischemic penumbra tissues showed that *Card19* mRNA levels increased 1 and 3 days after MCAO compared with the sham group. *n* = 3/group. (D, E) The representative western blot images and quantitative analysis of CARD19 expression after MCAO. *n* = 3/group. (F) Representative immunofluorescence images of CARD19 expression in microglia from the ischemic penumbra (TMEM119^+^) of mice 1 and 3 days after MCAO. Scale bar: 50 μm. (G) Percentage of CARD19-positive microglia. *n* = 4/group. (H) Representative Iba1 immunofluorescence staining images of mice were fed with PLX5622 or vehicle. Scale bar: 100 μm. (I) qPCR analysis showed that the increase in *Card19* expression was mainly produced by microglia after MCAO. *n* = 4/group. (J) Representative immunoblot images showing that LPS stimulation increased CARD19 expression in microglia. *n* = 4/group. (K) Representative immunoblot images showing that OGD/R stimulation increased CARD19 expression in microglia. *n* = 3/group. All experiments were repeated at least three times, with each using a separate brain or cell sample. Data are presented as mean ± SD. In C, E, and G, one-way analysis of variance followed by Bonferroni *post hoc* test was used. In I, two-way analysis of variance followed by Tukey’s *post hoc* test was used. In K, two-tailed unpaired Student’s *t-*test was used. *P < 0.05, **P < 0.01, ***P < 0.001. Card19: Caspase activation and recruitment domain 19; Iba1: ionized calcium binding adaptor molecule 1; LPS: lipopolysaccharide; MCAO: middle cerebral artery occlusion; OGD/R: oxygen–glucose deprivation/reoxygenation; qPCR: quantitative polymerase chain reaction; SD: standard deviation.

The data presented in **Figure 3A** are four coronal sections from the ischemic hemispheres of mice on day 3 after photothrombosis, and the data in **Figure 3B** are from a sham mouse and the ischemic hemispheres of mice at 3 hours, 1 day, and 3 days after MCAO (Li et al., 2023a; Han et al., 2024). The data shown in **Figure 3B** were obtained from Loupe Browser 7 software (10x Genomics, Pleasanton, USA) based on the 10x Genomics platform, and the data shown in **Figure 3A** were downloaded from the public website (http://www.jianglab.cn/StrokeMap/).

### Cell culture

Primary microglia were extracted from 1-day-old neonatal C57BL/6J mouse brains purchased from GemPharmatech, Nanjing, China (license No. SCXK (Su) 2018-0008). Whole brains were isolated under a stereomicroscope (AGONGYIQI, Yuyao, China) and digested with pancreatic enzyme at 37°C for 10 minutes. Digestion was terminated by adding primary microglia culture medium. Then, the digested tissue was centrifuged at 100 × *g* 4°C for 5 min to isolate single cells, which were used to inoculate tissue culture flasks. The first change of medium was performed 36–48 hours later, and the second change of medium was performed 6–7 days later. The primary microglia were collected after 10 days of incubation with shaking (200 r/min, 3 minutes). Primary microglia were culture in DMEM medium (Gibco, Suzhou, China, Cat# C11995500BT) with 10% FBS (Gibco, Cat#10099-141C) and 1% penicillin and streptomycin (Gibco, Cat#15140-122).

BV2 is an immortalized microglial cell line derived from mice that is extensively utilized in neuroscience investigations as an *in vitro* experimental model (Ye et al., 2020; Liu et al., 2023). BV2 cells (Pricella, Wuhan, China; Cat#CL-0493, RRID: CVCL_0182, accession number: CVCL_0182) were transfected with Card19 shRNA lentivirus (LV) or Flag-Card19 overexpression lentivirus for 72 hours and then treated with puromycin for 3 consecutive days. The culture condition used for the BV2 cells were the same as those described for primary microglia above.

### Lipopolysaccharide stimulation

BV2 cells were stimulated with lipopolysaccharide (LPS) (Sigma-Aldrich, Cat# HY-D1056; working concentration: 500 ng/mL) for 3 hours. Then, the BV2 cells were collected for subsequent experiments.

### Oxygen–glucose deprivation/reoxygenation

BV2 cells were placed in a sterilized modular incubator chamber (Billups-Rothenberg, Del Mar, CA, USA). The growth medium was removed, and the cells were washed with sterile 1×PBS three times. DMEM (glucose-free) (Gibco, Cat# 11966-025) was added to the cells, which were then placed in a chamber aerated with nitrogen mixed with 5% CO_2_ for 5–10 minutes and incubated at 37°C for 4 hours. After oxygen–glucose deprivation (OGD), the cells were removed from the chamber, the DMEM (glucose-free) was discarded, and fresh cell medium was added for reperfusion. Samples were collected for follow-up experiments after 24 hours of reperfusion.

### Western blotting

Brain tissues and BV2 cells were lysed with NP-40 lysis buffer (Beyotime, Shanghai, China) containing PMSF (Beyotime). Then, lysates containing equal amounts of protein were loaded into the wells of an SDS-PAGE gel (Epizyme, Shanghai, China) and subjected to electrophoresis, after which the proteins were transferred to PDVF membranes (Millipore, Billerica, MA, USA). The membranes were blocked with 5% nonfat milk for 1.5 hours at room temperature, then incubated with primary antibodies against CARD19 (rabbit, 1:1000, Abclonal, Wuhan, China, Cat# A13196), GAPDH (rabbit, 1:2000, Bioworld Technology, St. Louis Park, MN, USA, Cat# MB66349), β-actin (rabbit, 1:5000, Bioworld Technology, Cat# AP0060), mitochondrial inner membrane protein 19 (MIC19) (rabbit, dilution: 1:1000, Abclonal, Cat# A8584), mitochondrial inner membrane protein 60 (MIC60) (rabbit, 1:1000, Abclonal, Cat# A2751), sorting and assembly machinery component 50 (SAMM50) (rabbit, 1:1000, Abclonal, Cat# A3401), and Flag (rabbit, dilution: 1:1000, Abclonal, Cat# AE063) overnight at 4°C. The membranes were subsequently incubated with an HRP-conjugated secondary antibody (goat anti-rabbit, dilution: 1:5000, Bioworld Technology, Cat# BS22357) for 1.5 hours at room temperature, and the bands were visualized using an ECL chemiluminescence test kit (Vazyme, Nanjing, China, Cat# E411-04). Relative protein expression was normalized to β-actin and GAPDH. The bands were quantified using ImageJ (version 1.53o11).

### Co-immunoprecipitation

Firstly, protein A/G agarose beads (Beyotime) were washed with PBS three times. Then, 550 μg protein from BV2 cells was incubated with the pre-cleaned agarose beads at 4°C for 2 hours. Next, 50 μg protein was prepared as the input with 5× loading buffer. The remaining 500 μg protein was incubated with 1 μg MIC19 antibody (rabbit, 1:500, Abclonal, Cat# A8584) or Flag antibody (rabbit, 1:500, Abclonal, Cat# AE063) and IgG (rabbit, 1:500, Millipore, Cat# PP64B) at 4°C overnight and then incubated with the pre-cleaned agarose beads for 2 hours at 4°C. Finally, the agarose beads were washed five times with PBS. The proteins bound to the beads were eluted with 1× loading buffer and visualized by western blotting.

### Co-immunoprecipitation coupled with mass spectrometry

Co-immunoprecipitated proteins from BV2 cells were loaded into the wells of an SDS-PAGE gel, subjected to electrophoresis, and visualized by Coomassie Blue staining (SimplyBlue Safe Stain, Cat# LC6060, Thermo Fisher Scientific, Waltham, MA, USA). Mass spectrometry (MS) was performed by Oebiotech (Shanghai, China). Briefly, the protein strips were dissolved in Nano HPLC Buffer A after enzymatic hydrolysis and peptide desalination. A Nano-HPLC liquid phase system EASY-nLC1200 (Thermo Fisher Scientific) was used for separation, and a Q-Exactive mass spectrometer (Thermo Fisher Scientific) was used for mass spectrometry. The Uniprot Mus musculus database (https://www.uniprot.org/proteomes/UP000000589) was searched using MaxQuant (Max-Planck-Institute of Biochemistry, Martinsried, Germany) to identify the MS/MS spectra. CARD19-interacting proteins were identified using Metascape (https://metascape.org/).

### Immunofluorescence staining

The mice were anesthetized with a mixture of 1.5% isoflurane and 30% oxygen (RWD) at 1 and 3 days after MCAO and perfused transcardially with 30 mL PBS followed by 30 mL 4% PFA. The brains were collected and fixed in 4% PFA overnight, then transferred to 15% and 30% sucrose for 24 hours, and finally cryosectioned into 20-μm-thick slices. The slices were blocked with 2% BSA containing 0.25% Triton-X100 for 1 hour at room temperature, then incubated with primary antibodies against CARD19 (rabbit, 1:150, Thermo Fisher Scientific, Cat# PA5-89068), ionized calcium binding adaptor molecule (Iba1) (goat, 1:500, Abcam, Cambridge, UK, Cat# ab5076), TMEM119 (mouse, 1:500, Synaptic Systems, Goettingen, Germany, Cat# 400011), dsDNA (mouse, 1:500, Abcam, Cat# Ab27156), CD31 (mouse, 1:500, Abcam, Cat# ab64543), neuron-specific nuclear protein (NeuN; mouse, 1:500, Abcam, Cat# ab104224), glial fibrillary acidic protein (GFAP; mouse, 1:500, Cell Signaling Technology, Danvers, MA, USA, Cat# 3670), and heat shock protein 60 (HSP60; mouse, 1:500, Promab, Changsha, China, Cat# 30193) at 4°C overnight. The slices were washed three times with PBS and then incubated with secondary antibodies (donkey anti-mouse IgG (H+L) highly cross-adsorbed secondary antibody, Alexa Fluor^TM^ 488, Cat# A21202; donkey anti-Rabbit IgG (H+L) highly cross-adsorbed secondary antibody, Alexa Fluor^TM^ 488, Cat#21206; donkey anti-rabbit IgG (H+L) highly cross-adsorbed secondary antibody, Alexa Fluor^TM^ 594, Cat# 21207; donkey anti-rabbit IgG (H+L) highly cross-adsorbed secondary antibody, Alexa Fluor^TM^ Plus 647, Cat#32795; donkey anti-goat IgG (H+L) highly cross-adsorbed secondary antibody, Alexa Fluor^TM^ Plus 488, Cat# 32814; 1:500, Thermo Fisher Scientific) for 1.5 hours at room temperature. Nuclei were stained with 4′,6-diamidino-2-phenylindole (DAPI) (Cat#BD5010, Bioworld Technology) for 10 minutes. Finally, fluorescence images were taken using an Olympus FV3000 confocal microscope (Olympus, Tokyo, Japan). ImageJ (version 1.53o11) with the Skeletonize (2D/3D) plugin was used to quantify the process lengths, branches, end-point voxels, and junctions of the microglia. ImageJ (version 1.53o11) with the Colocalization Finder plugin was used to quantify co-localization between CARD19 and HSP60. A Pearson’s correlation coefficient value ranging from 0.5 to 1.0 was considered to indicate co-localization. Imaris (OXFORD INSTRUMENTS) was applied for 3D reconstruction.

### Terminal deoxynucleotidyl transferase dUTP nick end labeling staining

Terminal deoxynucleotidyl transferase dUTP nick end labeling (TUNEL) staining was conducted using a one-step TUNEL Apoptosis Assay Kit (Abbkine, Wuhan, China, Cat# KTA2010) according to the manufacturer’s instructions. Briefly, immunofluorescent staining for Iba1 was performed as described in section 1.16, followed by washing twice with 1×PBS for 5 minutes each and incubating in a 20 μg/mL proteinase K solution for 15 minutes. The samples were then washed three times with 1×PBS for 5 minutes each, treated with 100 μL of TdT labeling reaction buffer, and incubated in a humidified chamber at 37°C in the dark for 2 hours. After washing three times with 1×PBS for 5 minutes each, DAPI staining was performed, followed by three washes with 1×PBS for 5 minutes each. Finally, anti-fade reagent was added, and the slides were mounted.

### Mitochondrial superoxide indicator staining

The original cell culture mediumwas removed. Then, 1 mL mitochondrial superoxide indicator (MitoSOX) (Invitrogen, St. Louis Park, MN, USA, Cat# M36008) working solution (concentration: 5 μmol) was added to the BV2 cells, which were incubated at 37°C in the dark for 30 minutes. After washing with 1×PBS three times, the fluorescence intensity of mitochondrial ROS was observed using a fluorescence microscope (Olympus).

### Enzyme-linked immunosorbent assay

The concentrations of IL-1β, IL-6, and TNF-α in the ischemic penumbra in mice subjected to MCAO were measured using an enzyme-linked immunosorbent assay (ELISA) kit (Abclonal) according to the manufacturer’s protocol. The optical density (OD) value at 450 nm of each sample was measured using a Spark multi-function microplate detector (TECAN, Männedorf, Switzerland).

### Annexin V-PE/7-AAD apoptosis detection

Cell apoptosis was measured using an Annexin V-PE/7-AAD apoptosis detection kit (Vazyme) according to the manufacturer’s instructions. BV2 cells were rinsed with pre-cooled 1×PBS. Then, 100 μL 1×Binding Buffer was added to the cells, followed by 5 μL Annexin V-PE and 5 μL 7-AAD Staining Solution. The cells were incubated at room temperature in the dark for 10 minutes. Finally, 400 μL 1×Binding Buffer was added to the cells, and the signals were detected by flow cytometry (Accuri C6, BD Biosciences, Franklin Lake, NJ, USA).

### Quantitative polymerase chain reaction

Total mRNA was extracted from brain tissue and BV2 cells using FreeZol Reagent (Vazyme, R711-01) and reverse transcribed to cDNA using a PrimeScript RT Reagent Kit (Vazyme, R323-01) according to the manufacturer’s protocol. Quantitative polymerase chain reaction (qPCR) was performed using ChamQ Blue Universal SYBR qPCR Master Mix (Vazyme, Q321-02). The qPCR conditions were as follows: denaturation at 95°C for 30 seconds, followed by 45 cycles of 95°C for 5 seconds and 60°C for 30 seconds. The primer sequences are shown in **Additional Table 1**. Relative mRNA expression was normalized to β-actin.

**Additional Table 1:** Primers used for quantitative polymerase chain reaction analysis

Gene	Primer sequence (5'-3')
**IL-1β**	F: TGCCACCTTTTGACAGTGATG
	R: ATGTGCTGCTGCGAGATTTG
**TNF-α**	F: ATCTTCTCAAAATTCGAGTGAC
	R: TGGGAGTAGACAAGGTACAACCC
**IL-6**	F: CTAGGTTTGCCGAGTAGATCTC
	R: GACAAAGCCAGAGTCCTTCAGAGAG
**β-Actin**	F: AGATGTGGATCAGCAAGCAG
	R: GCGCAAGTTAGGTTTTGTCA
**GAPDH**	F: AGGTCGGTGTGAACGGATTTG
	R: GGGGTCGTTGATGGCAACA
**CARD19**	F: CCAGCCGCTACTCTCCTCA
	R: CGGTCAATGATGACAGGTG
**IFN-α**	F: AGTGAGCTGACCCAGCAGAT
	R: AGACAGCCTTGCAGGTCATT
**IFN-β**	F: CTGGCTTCCATCATGAACAA
	R: AGAGGGCTGTGGTGGAGAA
**D-loop**	F: AATCTACCATCCTCCGTGAAACC
	R: TCAGTTTAGCTACCCCCAAGTTTAA
**nDNA-Tert**	F: CTAGCTCATGTGTCAAGACCCTCTT
	R: GCCAGCACGTTTCTCTCGTT
**ND1**	F: CTAGCAGAAACAAACCGGGC
	R: CCGGCTGCGTATTCTACGTT

F: Forward; IFN-α: interferon-α; IFN-β: interferon-β; IL-1β: interleukin-ip; IL-6: interleukin-6; R: reverse; TNF-α: tumor necrosis factor-α.

### Quantitative polymerase chain reaction analysis of cytosolic mtDNA

Cytoplasmic DNA was extracted as previously described (Chung et al., 2019; He et al., 2022). Briefly, BV2 cells were collected, divided into two parts and then resuspended in buffer 1 (100 mM Tris-HCl, 5 mM EDTA, 0.2% SDS, 200 mM NaCl, 100 μg/mL protease K) for whole-cell extraction or buffer 2 (150 mM NaCl, 50 mM HEPES, 25 μg/mL digitonin) for cytoplasmic extraction. The cells were incubated with end-over-end mixing for 10 minutes at 4°C, then centrifuged at 17,000 × *g* for 25 minutes at 4°C. Whole-cell DNA and cytoplasmic DNA were extracted from the supernatants using a genomic DNA extraction kit (Beyotime). The qPCR of the whole-cell DNA and cytoplasmic DNA was performed using nuclear DNA (nDNA) primers targeting telomerase reverse transcriptase (Tert) and mtDNA primers targeting displacement loop (D-loop) and NADH dehydrogenase 1 (ND1). The CT values of nDNA abundance in the whole-cell extracts were normalized to the cytoplasmic mtDNA control. The primer sequences are listed in **Additional Table 1**.

### Transmission electron microscopy

After anesthesia, the mice were transcardially perfused with PBS and 4% electron microscope fixating solution. Next, a 1 mm × 1 mm × 1 mm cortical tissue sample surrounding the ischemic penumbra area was collected and immersed in electron microscope fixating solution at room temperature for 2 hours, and then transferred to 4°C and incubated overnight. Subsequent sectioning and electron microscopy were performed by Servicebio (Wuhan, China). Briefly, the sample was rinsed three times with 0.1 M phosphoric acid buffer (pH 7.0), then fixed with 1% osmic acid solution (SPI, West Chester, PA, USA) for 2 hours, dehydrated in an ethanol gradient (30%, 50%, 70%, and 80%) and an acetone gradient (90%, 95%, and 100%), and finally embedded. The sample was cut into 70- to 90-nm-thick slices using a LEICA EM UC7 ultra-thin microtome. The slices were stained with lead citrate solution for 10 minutes, then stained with a uranium dioxyacetate (SPI) 50% ethanol saturated solution for 10 minutes. The images were observed using an HT7800 transmission electron microscope (Hitachi High-Tech, Shanghai, China) at 80.0 kV and captured using a Hitachi TEM system (Hitachi, Tokyo, Japan). Mitochondria were divided into three categories based on the degree of mitochondrial damage: Class I, Class I, and Class III (Guo et al., 2023). The proportions of these three categories and the number of cristae junctions/mitochondrial cristae were quantified to evaluate the degree of mitochondrial structure damage.

### Mitochondrial membrane potential detection with tetramethylrhodamine ethyl ester

The original cell culture medium was removed, and the BV2 cells were washed once with 1×PBS. Then, 100 μL tetramethylrhodamine ethyl ester (TMRE) staining solution (Beyotime) was added, and the cells were incubated at 37°C for 30 minutes. The TMRE staining solution was removed, and the cells were washed once with 1×PBS. Finally, 100 μL preheated cell culture medium was added, and the fluorescence intensity was detected in Texas Red mode using a Spark multi-function microplate detector (TECAN, Switzerland).

### Fluoro-Jade B staining

Slides containing frozen brain sections from mice subjected to MCAO were dried for 30 minutes in a 50°C oven. The slides were then immersed in 70% alcohol for 2 minutes and transferred to distilled water for 2 minutes. Next, 0.06% potassium permanganate solution was added to the slides. After incubation for 10 minutes, the slides were washed with distilled water for 2 minutes and then incubated in a 0.1% acetic acid (Zhanyun, Shanghai, China) solution containing 0.001% Fluoro-Jade B (FJB) (Biosensis, Thebarton, South Australia, Cat# TR-150-FJB) for 10 minutes. The slides were rinsed three times with distilled water for 1 minute each, air-dried in a 50°C oven for 5 minutes, soaked in xylene for 1 minute, and then sealed with neutral resin. The number of FJB-positive cells per mm^2^ was determined by confocal microscopy (Olympus). For each sample, the infarct area was photographed, and the number of FJB-positive cells per mm^2^ was calculated using ImageJ. The result was obtained by averaging the number of FJB-positive cells per mm^2^ in four fields from each sample.

### Statistical analysis

GraphPad Prism 8.0 software (GraphPad Software, San Diego, CA, USA, www.graphpad.com) was used for statistical analysis. All data are presented as mean ± standard deviation (mean ± SD), except for ordinal categorical variables, which are presented as median and interquartile range (IQR). For comparisons between two groups, Student’s *t*-test was used when continuous variables were normally distributed; otherwise, the Mann–Whitney *U* test was used. For comparisons of three or more groups, one-way analysis of variance followed by Bonferroni *post hoc* test was performed for normally distributed data. For comparisons between two factors and two groups, two-way analysis of variance followed by Tukey’s *post hoc* test was performed on normally distributed data. Mann–Wilcoxon rank sum test was used for ordinal categorical variables. *P* < 0.05 was considered statistically significant.

## Results

### Microglial CARD19 expression is increased in the ischemic penumbra

We and others have recently performed spatial transcriptomic sequencing of ischemic brain tissue collected from MCAO mice (Li et al., 2023a; Han et al., 2024). After reanalyzing the published data, we found that *Card19* expression was markedly increased in microglia 3 hours and 12 hours after MCAO compared with the sham group. Notably, the spatial transcriptomic results showed that the elevated *Card19* expression was mainly distributed in the ischemic penumbra (**Figure 3A** and **B**).

qPCR and western blotting confirmed that Card19 mRNA and protein levels were significantly increased in the ischemic penumbra at 1 and 3 days after MCAO (**Figure 3C–E**). Furthermore, immunofluorescence staining showed that compared with the sham group, microglial CARD19 expression was increased 1 and 3 days after MCAO (**Figure 3F** and **G**). To determine whether microglia were responsible for the elevated *Card19* expression levels, mice were fed 8 g feed containing PLX5622 (a CSF1R inhibitor) daily for 2 weeks to deplete microglia. The efficiency of microglial depletion was verified by immunofluorescence staining (**Figure 3H**). The results showed that, the elevated *Card19* mRNA levels observed after MCAO decreased significantly to physiological levels after microglia depletion (MCAO1d + Vehicle *vs.* MCAO1d + PLX5622, *P* = 0.0081), indicating that the increased CARD19 expression seen after MCAO was mainly derived from microglia (**Figure 3I**).

Since DAMPs can act on microglial Toll-like receptors to initiate a pro-inflammatory response after ischemic stroke, we stimulated cells with LPS *in vitro* to mimic microglial activation during the acute phase of ischemic stroke. The results showed that treating primary microglia with LPS increased CARD19 expression compared with the control group (**Figure 3J**). Moreover, when oxygen–glucose deprivation/reoxygenation (OGD/R) was applied to microglia to mimic oxygen and energy deficiency after ischemic stroke, CARD19e protein levels increased compared with the control group (**Figure 3K**). Taken together, these findings suggest that microglial CARD19 expression increases in the penumbra after ischemic stroke.

### Microglial CARD19 deficiency aggravates ischemic brain injury

**Figure 4:**
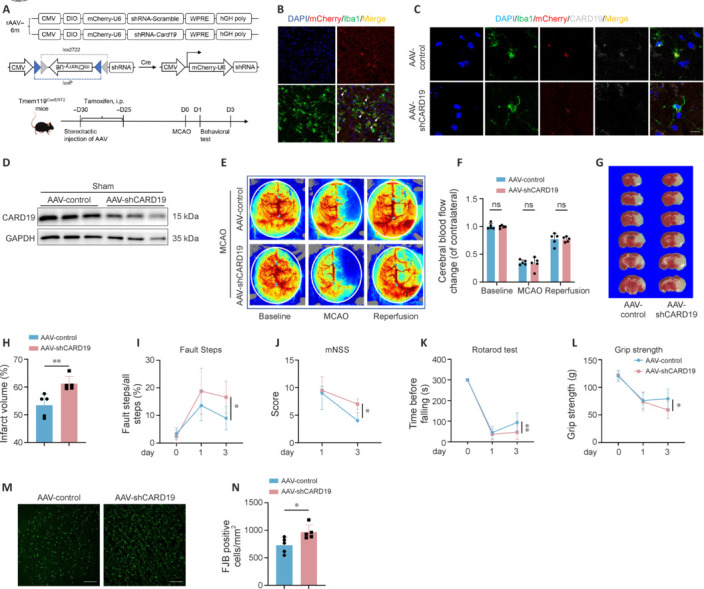
Microglial CARD19 deficiency aggravated ischemic brain injury. (A) AAV design and time plot of stereotaxic injection. (B) Representative immunofluorescence images of microglia (Iba1) efficiently transfected with AAV. Scale bar: 50 μm. (C) Representative immunofluorescence images of DAPI, microglia (green), AAV (red), and CARD19 (gray). Scale bar: 10 μm. (D) Western blot results showing that CARD19 expression was decreased in AAV-shCARD19–injected mice compared with AAV-control–treated mice. *n* = 3/group. (E) Representative CBF images were obtained 10 minutes before MCAO (baseline), 10 minutes after surgery, and 30 minutes after reperfusion. White circles: regions of interest. (F) Quantitative analysis of regional CBF. *n* = 5/group. (G, H) Representative images of TTC staining and quantification of infarct volume in AAV-control– and AAV-shCARD19–injected mice at days after MCAO. Normal tissues stained red, and infracted tissues remained white. *n* = 5/group. (I–L) Foot fault test (I), mNSS test (J), rotarod test (K), and grip strength (L) in AAV-control– and AAV-shCARD19–injected mice 1 and 3 days after MCAO. (M, N) Representative immunofluorescence images of FJB staining and quantitative analysis showed neuronal death in AAV-control– and AAV-shCARD19–injected mice 3 days after MCAO. *n* = 5/group. All experiments were repeated at least three times, with each using a separate brain sample. Data are presented as mean ± SD, except in Figure J, where data are presented as median and IQR. In F, I, K, L, two-way ANOVA followed by Tukey’s *post hoc* test was used. In J, Mann–Wilcoxon rank sum test was used. In H, N, two-tailed unpaired Student’s *t*-test was used. *P < 0.05, **P < 0.01. AAV: Adeno-associated virus; CARD19: caspase activation and recruitment domain 19; CBF: cerebral blood flow; D: day; DAPI: 4′,6-diamidino-2-phenylindole; FJB: Fluoro-Jade B; Iba1: ionized calcium binding adaptor molecule 1; IQR: interquartile range; MCAO: middle cerebral artery occlusion; mNSS: modified neurological severity scores; SD: standard deviation; TTC: 2,3,5-triphenyltetrazolium chloride.

To investigate the role of microglial CARD19 in ischemic stroke, we conditionally knocked down *Card19* expression in microglia through stereotaxic injection of AAVs into the cerebral cortex of Tmem119^Cre/ERT2^ mice (**Figure 4A**). Immunofluorescence staining showed that microglia were efficiently transfected, and CARD19 knockdown was successful (**Figure 4B–D** and **Additional Figure 1D–F**). Co-staining for AAV and other cell types showed that the majority of AAV co-localized with microglia, while only a minimal amount of AAV was detected in astrocytes. There was no significant co-localization of AAV with endothelial cells (CD31) or neurons (NeuN) (**Additional Figure 1A–C**). These findings suggested that AAV transfected microglia and specifically knocked down CARD19. Next, mice in the AAV-control group and AAV-shCARD19 group were subjected to MCAO. There are no significant differences in regional CBF between AAV-control and AAV-shCARD19 mice at minutes before MCAO (baseline), 10 minutes after surgery, or 30 minutes after reperfusion (**Figure 4E** and **F**). TTC staining showed that the infarct volume of AAV-shCARD19 mice was larger than that of AAV-control mice (**Figure 4G** and **H**). A series of behavioral tests (foot fault test, mNSS test, rotarod test, and grip strength test) showed that the AAV-shCARD19 mice exhibited more severe neurological deficits than the AAV-control mice (**Figure 4I–L**). Fluoro-Jade B staining showed that more neurons died in AAV-shCARD19 mice than in AAV-control mice (**Figure 4M** and **N**). These results suggested that conditional knockdown of CARD19 in the microglia aggravates brain injury after ischemic stroke.

### Microglial CARD19 deficiency amplified neuroinflammation in ischemic stroke

**Figure 5:**
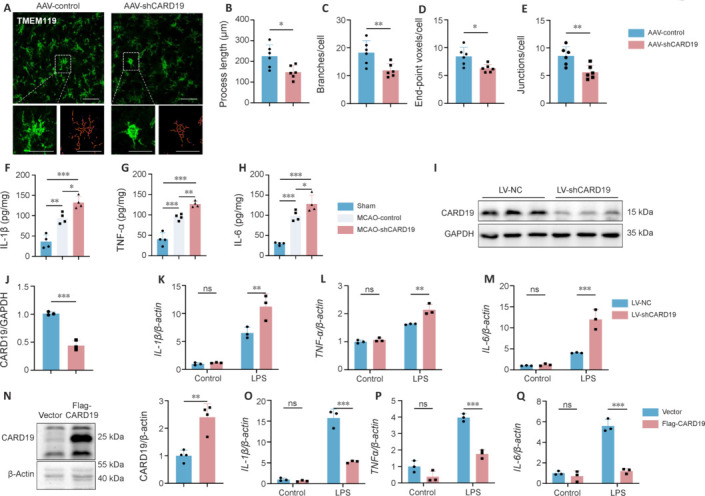
Microglial CARD19 deficiency amplifies post-stroke neuroinflammatio. (A) Representative immunofluorescence images of microglia (TMEM119^+^) in AAV-control– and AAV-shCARD19–injected mice 3 days after MCAO. Scale bars: 100 μm (upper) and 50 μm (lower). (B–E) Quantification of process length (B), branches (C), end-point voxels (D), and junctions (E) in microglia in AAV-control and AAV-shCARD19 mice. *n* = 6/group. (F–H) Enzyme-linked immunosorbent assay of pro-inflammatory cytokines including IL-1β (F), TNF-α (G), and IL-6 (H) in AAV-control– and AAV-shCARD19–injected mice 3 days after MCAO. *n* = 4/group. (I, J) Western blot analysis showing that CARD19 was efficiently knocked down in BV2 cells. *n* = 3/group. (K–M) qPCR analysis of the expression of pro-inflammatory cytokines including *IL-1*β (K), *TNF-α* (L), and *IL-6* (M) in LV-NC and LV-shCARD19 microglia stimulated with LPS for 3 hours. *n* = 3/group. (N) Western blot showing that CARD19 was efficiently overexpressed in BV2 cells. (O–Q) qPCR analysis of the expression of pro-inflammatory cytokines including *IL-1*β (O), *TNF-α* (P), and *IL-6* (Q) in Vector and Flag-CARD19 microglia stimulated with LPS for 3 hours. *n* = 3/group. All experiments were repeated at least three times, with each using a separate brain or cell sample. Data are presented as mean ± SD. In F–H, one-way ANOVA followed by Bonferroni *post hoc* test was used. In K–M, O–Q, two-way ANOVA followed by Tukey’s *post hoc* test was used. In B–E, J, and N two-tailed unpaired Student’s *t*-test was used. *P < 0.05, **P < 0.01, ***P < 0.001. AAV: Adeno-associated virus; CARD19: caspase activation and recruitment domain 19; IL-1β: interleukin-1β; IL-6: interleukin-6; LPS: lipopolysaccharide; LV: lentivirus; MCAO: middle cerebral artery occlusion; qPCR: quantitative polymerase chain reaction; SD: standard deviation; TNF-α: tumor necrosis factor-α.

Since microglia plays a pro-inflammatory role during the acute phase of ischemic stroke, we next asked whether microglial CARD19 deficiency affects neuroinflammation post-stroke. Homeostatic microglia have small cell bodies and ramified branches. After ischemic stroke, microglia are activated rapidly and adopt an amoeboid-like phenotype (Jia et al., 2021). We therefore observed microglial morphology in the ischemic penumbra by TMEM119 immunofluorescence staining. Microglia in the AAV-shCARD19 mice exhibited larger cell bodies and shorter and less ramified branches than microglia in the AAV-control mice on the 3rd day after MCAO (AAV-control *vs*. AAV-shCARD19: process length, *P* = 0.0150; branches, *P* = 0.0099; end-point voxels, *P* = 0.0129; junctions, *P* = 0.0060; **Figure 5A–E**). Furthermore, compared with AAV-control mice, AAV-shCARD19 mice exhibited higher levels of pro-inflammatory factors, including IL-1β, TNF-α, and IL-6 (IL-1β: Sham *vs.* MCAO-control, *P* = 0.0011; Sham *vs*. MCAO-shCARD19, *P* < 0.0001; MCAO-control *vs*. MCAO-shCARD19, *P* = 0.0142; TNF-α: Sham *vs*. MCAO-control, *P* = 0.0002; Sham *vs*. MCAO-shCARD19, *P* < 0.0001; MCAO-control *vs.* MCAO-shCARD19, *P* = 0.0066; IL-6: Sham *vs*. MCAO-control, *P* = 0.0002; Sham *vs.* MCAO-shCARD19, *P* < 0.0001; MCAO-control *vs.* MCAO-shCARD19, *P* = 0.0435; **Figure 5F–H**). To further investigate the role of CARD19 in microglia, we knocked down or overexpressed CARD19 in BV2 cells using lentiviruses (**Figure 5I** and **J**) and found that the levels of *IL-1*β, *TNF-α*, and *IL-6* mRNA in LV-shCARD19-treated microglia were significantly higher than those in LV-NC-treated microglia after LPS stimulation (*IL-1*β/β*-actin*: LV-NC + LPS *vs.* LV-shCARD19 + LPS, *P* = 0.0074; *TNF-α*/β*-actin*: LV-NC + LPS *vs.* LV-shCARD19 + LPS, *P* = 0.0020; *IL-6*/β*-actin*: *P* = 0.0002; **Figure 5K–M**). Consistent with this, CARD19 overexpression suppressed *IL-1*β, *TNF-α*, and *IL-6* mRNA levels after LPS stimulation (**Figure 5N–Q**). Thus, CARD19 deficiency promotes microglial activation and amplifies neuroinflammation in ischemic stroke.

### Microglial CARD19 deficiency accelerates ultrastructural and functional damage to mitochondria in ischemic stroke

**Figure 6:**
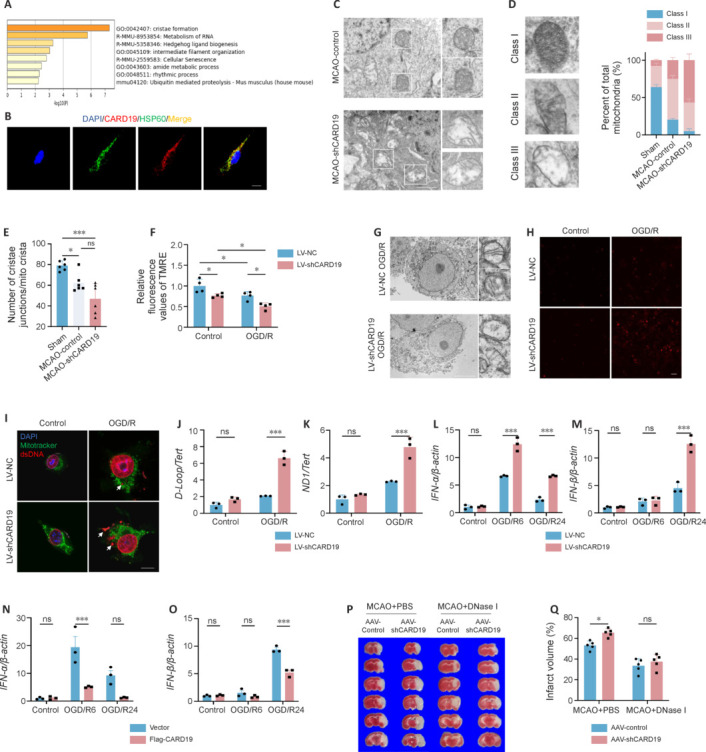
Microglial CARD19 deficiency accelerates ultrastructural and functional damage to mitochondria in ischemic stroke. (A) IP-MS analysis of CARD19-interacting proteins. (B) Representative immunofluorescence images of HSP60 (green), CARD19 (red), and DAPI (blue) in primary microglia. Scale bar: 5 μm. (C) Representative transmission electron microscope image of mitochondria in microglia from AAV-control– and AAV-shCARD19–injected mice 3 days after MCAO. (D) Quantification of Class I, Class II, and Class III mitochondria in AAV-control– and AAV-shCARD19–injected mice. *n* = 3/group. (E) Quantification of cristae junctions/mitochondrial cristae in AAV-control– and AAV-shCARD19–injected mice. *n* = 6/group. (F) Relative TMRE fluorescence values in LV-NC– and LV-shCARD19–treated microglia after OGD/R. (G) Representative transmission electron microscope images of mitochondria in LV-NC– and LV-shCARD19–treated microglia after OGD/R. (H) Representative immunofluorescence images of MitoSOX in LV-NC– and LV-shCARD19–treated microglia after OGD/R. Scale bar: 100 μm. (I) Representative immunofluorescence images of mitotracker (green), dsDNA (red), and DAPI (blue) in LV-NC– and LV-shCARD19–treated microglia after OGD/R. Scale bar: 10 μm. (J, K) qPCR analysis of *D-Loop/Tert* (J) and *ND1/Tert* (K) in LV-NC– and LV-shCARD19–treated microglia after OGD/R. *n* = 3/group. (L, M) qPCR analysis of *IFN-α* (L) and *IFN-*β (M) in LV-NC– and LV-shCARD19–treated microglia after OGD/R. *n* = 3/group. (N, O) qPCR analysis of *IFN-α* (N) and *IFN-*β (O) in Vector and Flag-CARD19/group microglia after OGD/R. *n* = 3/group. (P, Q) Representative images of TTC staining and quantitative analysis of AAV-control– and AAV-shCARD19–injected mice 3 days after MCAO injected with PBS or DNase I. Normal tissues stained red, and infracted tissues remained white. *n* = 4/group. All experiments were repeated at least three times, with each using a separate brain or cell sample. Data are presented as mean ± SD. In E, one-way ANOVA followed by Bonferroni *post hoc* test was used. In F, J–O, Q, two-way ANOVA followed by Tukey’s *post hoc* test was used. *P < 0.05, ***P < 0.001. AAV: Adeno-associated virus; ANOVA: analysis of variance; CARD19: caspase activation and recruitment domain 19; DAPI: 4′,6-diamidino-2-phenylindole; D-loop: displacement loop; HSP60: heat shock protein 60; IFN-α: interferon-α; IFN-β: interferon-β; LV: lentivirus; MCAO: middle cerebral artery occlusion; MitoSOX: mitochondrial superoxide indicator; ND1: NADH dehydrogenase 1; ns: not significant; OGD/R: oxygen–glucose deprivation/reoxygenation; qPCR: quantitative polymerase chain reaction; SD: standard deviation; Tert: telomerase reverse transcriptase; TMRE: tetramethylrhodamine, ethyl ester; TTC: 2,3,5-triphenyltetrazolium chloride.

To investigate the mechanism underlying the neuroprotective role of microglial CARD19, we screened for CARD19-interacting proteins by immunoprecipitation-mass spectrometry. The proteins that interacted with CARD19 in microglia were enriched in the mitochondrial cristae formation pathway (**Figure 6A**). Immunofluorescence staining confirmed that CARD19 co-localized with HSP60 (a marker of mitochondria) in primary microglia (Pearson’s_Rr = 0.84222891), indicating that CARD19 may play a functional role in the mitochondria of microglia (**Figure 6B** and **Additional Figure 2**).

When ischemic stroke occurs, cerebral blood flow is blocked, resulting in microglial mitochondria dysfunction, which manifests as reduced ATP production, energy metabolism disorders, and mitochondrial structural damage (Li et al., 2014; Yang et al., 2018). To investigate the role of CARD19 in the maintenance of mitochondrial cristae in microglia, the mitochondrial ultrastructure of microglia in the ischemic penumbra on day 3 after MCAO was observed by transmission electron microscopy (**Figure 6C**). Our results showed that, after MCAO, the proportion of Class I mitochondria was significantly reduced from 60%–70% to 20%, that of Class II mitochondria was significantly increased from 20%–30% to 50%–60%, and that of Class III mitochondria was significantly increased from 10% to 20%–30% compared with sham controls (Sham *vs*. MCAO-control: Class I, *P* < 0.0001; Class II, *P* < 0.0001; Class III: *P* < 0.0001). However, the proportion of Class I mitochondria was significantly reduced to around 10%, that of Class II mitochondria was reduced 30%–40%, and that of Class III mitochondria was increased to 50%–70% in AAV-shCARD19-treated mice compared with AAV-control-treated MCAO mice (MCAO-control *vs.* MCAO-shCARD19: Class I, *P* = 0.0079; Class II, *P* = 0.0050; Class III: *P* < 0.0001; **Figure 6D**). Additionally, after MCAO, the ratio of cristae junctions/mitochondrial cristae was decreased in AAV-shCARD19-treated mice compared with AAV-control-treated mice, suggesting an increasing in cristae disintegration (Sham *vs*. MCAO-control, *P* = 0.0361; Sham *vs*. MCAO-shCARD19, *P* = 0.0002; MCAO-control *vs.* MCAO-shCARD19, *P* = 0.0539; **Figure 6E**). Moreover, the results from electron microscopy analysis of OGD/R-treated microglia cells were consistent with those obtained in mice (**Figure 6G**). These findings suggest that microglial CARD19 deficiency exacerbates mitochondrial structural damage after ischemic stroke.

After ischemic stroke, hypoxia induces decoupling of the mitochondrial respiratory chain, leading to a surge in superoxide ROS production in mitochondria, as well as a decline in the mitochondrial membrane potential (An et al., 2021). Mitochondrial oxidative stress subsequently disrupts mtDNA integrity and promotes the release of oxidized mtDNA into the cytoplasm (Kim et al., 2023). Therefore, we examined relevant indicators of mitochondrial dysfunction. Compared with LV-NC-treated microglia, mitochondrial ROS production and mtDNA release were increased in the LV-shCARD19 group (D-Loop/Tert: LV-NC + OGD/R *vs*. LV-shCARD19 + OGD/R, *P* < 0.0001; ND1/Tert: LV-NC + OGD/R *vs*. LV-shCARD19 + OGD/R, *P* = 0.0003; **Figure 6H–K**). In addition, the mitochondrial membrane potential was decreased in LV-shCARD19-treated mice compared with LV-NC-treated microglia (LV-NC + Control *vs*. LV-shCARD19 + Control, *P* = 0.0393; LV-NC + Control *vs*. LV-NC + OGD/R, *P* = 0.0404; LV-shCARD19 + Control *vs*. LV-shCARD19 + OGD/R, *P* = 0.0255; LV-NC + OGD/R *vs*. LV-shCARD19 + OGD/R, *P* = 0.0248; **Figure 6F**). These findings suggest that microglial CARD19 deficiency inhibits mitochondrial function under OGD/R conditions.

Previous studies have shown that disruption of mitochondrial cristae leads to mtDNA release and mtDNA-dependent interferon I responses (He et al., 2022). To determine whether microglial CARD19 deficiency–induced mtDNA release activates the IFN-I response, we examined IFN-α and IFN-β mRNA levels by qPCR and found that CARD19 knockdown boosted the OGD/R-induced increase in IFN-α and IFN-β expression (**Figure 6L** and **M**). Conversely, CARD19 overexpression abolished the OGD/R-induced IFN-I response (**Figure 6N** and **O**). Next, we depleted cytoplasmic mtDNA by intraperitoneal injection of DNase I. TTC staining showed that mtDNA clearance partially reversed the adverse outcomes caused by microglial CARD19 deficiency (**Figure 6P** and **Q**). These results suggested that microglial CARD19 deficiency aggravates brain injury by promoting mitochondrial damage and provoking mtDNA-dependent interferon I responses.

**Figure 7:**
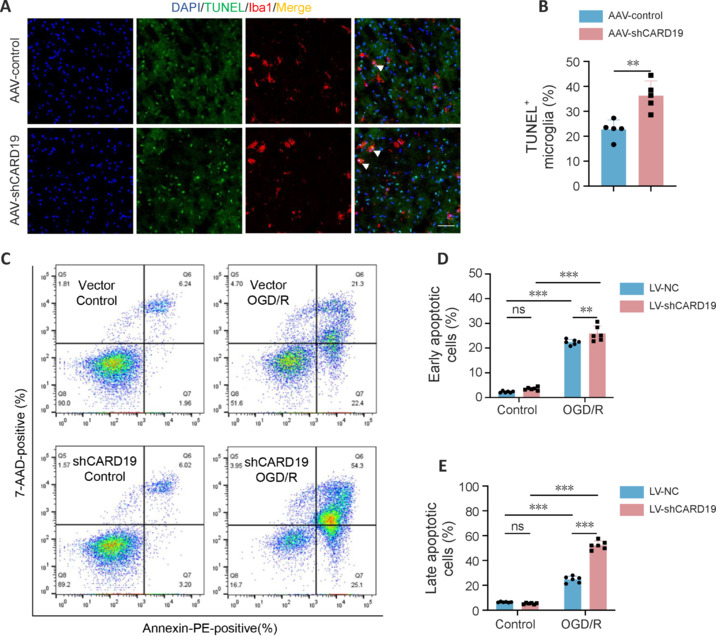
Microglial CARD19 deficiency accelerates apoptosis in ischemic stroke. (A) Representative immunofluorescence images of TUNEL (green), Iba1 (red), and DAPI (blue) in AAV-control– and AAV-shCARD19–injected mice 3 days after MCAO. Scale bar: 100 μm. (B) Quantification of TUNEL^+^ microglia in AAV-control– and AAV-shCARD19–injected mice. *n* = 5/group. (C) Representative flow cytometric analysis of apoptosis in LV-NC– and LV-shCARD19–treated microglia after OGD/R. (D, E) Quantification of early (D) and late (E) apoptotic cells in LV-NC– and LV-shCARD19–treated microglia after OGD/R. *n* = 6/group. All experiments were repeated at least three times, with each using a separate brain or cell sample. Data are presented as mean ± SD. In D, E, two-way analysis of variance followed by Tukey’s *post hoc* test was used. In B, two-tailed unpaired Student’s *t*-test was used. **P < 0.01, ***P < 0.001. AAV: Adeno-associated virus; CARD19: caspase activation and recruitment domain 19; DAPI: 4′,6-diamidino-2-phenylindole; Iba1: ionized calcium binding adaptor molecule 1; LV: lentivirus; MCAO: middle cerebral artery occlusion; ns: not significant; OGD/R: oxygen–glucose deprivation/reoxygenation; SD: standard deviation; TUNEL: terminal deoxynucleotidyl transferase dUTP nick end labeling.

Mitochondrial dysfunction, along with increased ROS production and calcium overload, occur after ischemic stroke, which results in opening of the mitochondrial membrane permeability transition pore, release of cytochrome c, and consequent activation of effector caspases, ultimately leading to apoptosis (Sims and Muyderman, 2010). Thus, we investigated the effect of microglial CARD19 deficiency on apoptosis *in vivo*. TUNEL staining showed that, compared with AAV-control-treated mice, the proportion of TUNEL^+^Iba1^+^ cells in Iba1^+^ cells was significantly reduced in AAV-shCARD19-treated mice (**Figure 7A** and **B**). Next we detected apoptosis of cultured microglia after OGD/R in both LV-NC- and LV-shCARD19-treated microglia using an Annexin V-PE/7-AAD apoptosis detection kit and found that, compared with LV-NC–treated microglia, the Annexin-V (+) 7-AAD (–) and Annexin-V (+) 7-AAD (+) ratios were significantly increased in LV-shCARD19-treated microglia (early apoptosis: LV-NC + Control *vs*. LV-NC + OGD/R, *P* < 0.0001; LV-shCARD19 + Control *vs.* LV-shCARD19 + OGD/R, *P* < 0.0001; LV-NC + OGD/R *vs.* LV-shCARD19 + OGD/R, *P* = 0.0080. Late apoptosis: LV-NC + Control *vs*. LV-NC + OGD/R, *P* < 0.0001; LV-shCARD19 + Control *vs*. LV-shCARD19 + OGD/R, *P* < 0.0001; LV-NC + OGD/R *vs*. LV-shCARD19 + OGD/R, *P* < 0.0001; **Figure 7C–E**). These findings suggested that CARD19 deficiency aggravates microglial apoptosis after brain ischemia. Therefore, microglial CARD19 deficiency aggravates the destruction of mitochondrial cristae, leading to mitochondrial dysfunction in ischemic stroke.

### CARD19 interacted with MIB components and stabilized mitochondrial cristae

**Figure 8:**
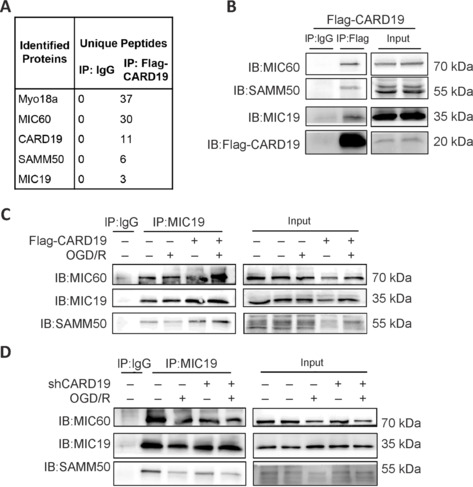
CARD19 interacts with MIB components and stabilizes mitochondrial cristae. (A) IP-MS results showing that CARD19 interacts with Myo18a, MIC60, SAMM50, and MIC19. (B) Representative immunoblot images showing that CARD19 interacts with MIC60, SAMM50, and MIC19 in microglia. (C) Representative immunoblot images showing that CARD19 overexpression partially rescued the binding between MIC19 and SAMM50 after OGD/R in microglia. (D) Representative immunoblot images showing that CARD19 knockdown of exacerbated the loss of binding between MIC19 and SAMM50 after OGD/R in microglia. All experiments were repeated at least three times, with each using a separate cell sample. CARD19: Caspase activation and recruitment domain 19; IP-MS: immunoprecipitation-mass spectrometry; MIB: mitochondrial intermembrane bridge; MIC19: mitochondrial inner membrane protein 19; MIC60: mitochondrial inner membrane protein 60; OGD/R: oxygen–glucose deprivation/reoxygenation; SAMM50: sorting and assembly machinery component 50.

IP-MS showed that core components of the MIB (e.g., MIC60, SAMM50, and MIC19) may interact with CARD19 in microglia. We verified these interactions by co-immunoprecipitation (**Figure 8A** and **B**), showing that CARD19 is able to bind MIB components. The mitochondrial intermembrane bridge is pivotal for mitochondrial cristae architecture and efficient oxidative phosphorylation (Zick et al., 2009). Breakdown of the connections among SAMM50, MIC19, and MIC60 leads to the loss of mitochondrial cristae connections and abnormal cristae distribution (Tang et al., 2020b). To test whether CARD19 participates in SAMM50-MIC19-MIC60 assembly, co-immunoprecipitation was performed. Our results showed that the interaction between SAMM50 and MIC19 in microglia was decreased after OGD/R. Binding between MIC19 and MIC60 was not affected by OGD/R. Importantly, CARD19 overexpression partially rescued the binding between MIC19 and SAMM50 after OGD/R, while CARD19 knockdown exacerbated the loss of binding between MIC19 and SAMM50 after OGD/R (**Figure 8C** and **D**). These results indicate that microglial CARD19 promotes MIB assembly, which may stabilize mitochondrial cristae in microglia.

## Discussion

In the current study, we explored the role and underlying mechanism of microglial CARD19 in ischemic stroke. CARD19 expression was increased in microglia after ischemic stroke. Microglial CARD19 deficiency exacerbated microglial activation, neuroinflammation, and ischemic brain injury, leading to mitochondrial cristae disruption and mitochondrial dysfunction. Mechanistically, microglial CARD19 stabilized mitochondrial cristae by facilitating the assembly of MIB components.

Previous studies have reported CARD19 expression in T cells, B cells, and macrophages (Suzuki et al., 2019; Rios et al., 2020; Bjanes et al., 2021; Wang et al., 2022; Zheng et al., 2023). However, there is no evidence for alterations in CARD19 expression in the context of disease or in response to pathological stimuli. We found that CARD19 expression was mainly elevated in microglia during the acute phase of ischemic stroke and primarily distributed in the ischemic penumbra. CARD19 expression was also elevated in microglia OGD/R or stimulation with LPS *in vitro*. It is worth noting that, although our results verified that CARD19 was primarily upregulated in microglia after MCAO, CARD19 function in infiltrating neutrophils and macrophages after ischemic stroke should be studied more comprehensively in the future.

Next, we found that microglial CARD19 deficiency amplified neuroinflammation in ischemic stroke. CARD19 belongs to the caspase activation and recruitment domain protein family that is broadly involved in the regulation of immune responses, apoptosis, and inflammation (Park, 2019). For example, NOD-like receptor 2 mediates inflammation through the MAPK/NF-κB signaling pathway, thereby aggravating brain damage, after ischemic stroke (Li et al., 2010; Liu et al., 2015). In MCAO mice, microglial TREM-1 expression activates the downstream CARD9/NF-κB pro-inflammatory pathway, which contributes to post-stroke neuroinflammatory damage (Xu et al., 2019). Conversely, CARD8 negatively regulates caspase1-dependent IL-1β production and inhibits NF-κB activation (Taabazuing et al., 2020). Hence, despite all members of the CARD protein family containing a CARD domain, they have two opposing functions, with certain members facilitating pro-inflammatory responses and others attenuating inflammation. CARD19 was first identified as a novel BCL10-interacting CARD protein that potently suppressed Bcl10-induced NF-κB activation, suggesting that CARD19 may play a role in inhibiting inflammation (Woo et al., 2004). In addition, a recent study revealed that CARD19 can inhibit NF-κB activation when transiently overexpressed in HEK293T cells; however, the absence of endogenous CARD19 expression had little effect on Bcl10-dependent NF-κB activation after TCR engagement in primary murine CD8 T cells (Rios et al., 2020). Our findings suggest that endogenous CARD19 deficiency exaggerates the microglia-mediated pro-inflammatory response both *in vivo* and *in vitro*. In contrast, CARD19 overexpression inhibited microglia-mediated inflammatory responses. The inflammatory cascade that activated during the acute phase of ischemic stroke promotes cell death and exacerbates tissue injury (Jayaraj et al., 2019; Wang et al., 2023). Increasing evidence shows that targeted inhibition of pro-inflammatory mediators can alleviate the inflammatory response and improve outcomes in ischemic stroke (Cai et al., 2017; Lin et al., 2021; Yuan et al., 2021). Thus, our findings suggest a novel therapeutic target to suppress microglia-mediated neuroinflammation after ischemic stroke.

Furthermore, we found that CARD19 deficiency destroyed the mitochondrial ultrastructure in microglia, especially mitochondrial cristae morphology. Microglial CARD19 deficiency promoted mitochondrial dysfunction in ischemic stroke. Specifically, CARD19 knockdown in microglia significantly increased mitochondrial ROS generation, increased mtDNA translocation into the cytosol, and induced marked depolarization of the mitochondrial membrane. Moreover, CARD19 deficiency induced mtDNA-dependent interferon I responses in microglia. mtDNA clearance reversed the increase in infarct volume caused by microglial CARD19 deficiency. Mitochondrial dysfunction occurs within minutes after brain ischemia and is considered the determining event of ischemic stroke. Maintaining normal mitochondrial function is essential for cell survival after ischemic stroke. Mitochondrial dysfunction induced by hypoxia and glucose deprivation occurs within minutes after ischemia, leading to a decrease in ATP production and overproduction of ROS (Sims and Muyderman, 2010). The intricate interplay between mitochondrial dysfunction and inflammation has a substantial impact on microglial function. High concentrations of Ca^2+^ in mitochondria promote sustained opening of mitochondrial permeability transition pore (MPTP) and enhance the permeability of the mitochondrial inner membrane (Hurst et al., 2017). Various mitochondrial components and products are released as a result of mitochondrial dysfunction (and potential cell death) that aggravate the inflammatory response (Marchi et al., 2023). Mitochondrial ROS induce the generation of oxidized mitochondrial DNA (ox-mtDNA), which is cleaved by Flap endonuclease 1 and escapes through the MPTP into the cytosol, where ox-mtDNA binds to NLRP3 and cyclic GMP-AMP synthase, which collectively initiate the inflammatory responses (Shimada et al., 2012; Xian et al., 2022). Moreover, mtDNA and ROS can activate the inflammasome and subsequently drive IL-1β and IL-18 secretion (Zhang et al., 2010; Nakahira et al., 2011; Zhou et al., 2011). MtDNA can also engage Toll-like receptor 9, activating the NF-κB signaling pathway and promoting the transcription of pro-inflammatory cytokines (Mills et al., 2017). Recent evidence suggests that mtDNA is involved in neuroinflammation after ischemic brain injury. The absence of cyclic GMP-AMP synthase production by microglia mitigates the neuroinflammation induced by ischemia-reperfusion injury (Liao et al., 2020). Moreover, mitochondria, as the powerhouses of the cell, are pivotal for brain energy metabolism. In the resting state, microglia predominantly utilize oxidative phosphorylation to produce ATP, while during inflammation or stress, microglia rapidly upregulate glycolysis to offset energy deficits (Wang et al., 2019; Preeti et al., 2022). Inhibition of oxidative phosphorylation and the electron transport chain activates microglia, resulting in elevated mitochondrial ROS production, NLRP3 inflammasome activation, pro-inflammatory cytokine release, mitochondrial injury, and subsequent microglial apoptosis (Shaikh and Nicholson, 2009; Ye et al., 2016). Reprogramming microglial energy metabolism from glycolysis to oxidative phosphorylation enhances phagocytic clearance of neutrophils by microglia following ischemic stroke (Li et al., 2023b). Our findings indicate that CARD19 deficiency contributes to the disruption of mitochondrial cristae, decreases the mitochondrial membrane potential, increases ROS generation, and enhances mtDNA release into the cytosol. Future studies should explore whether altered glucose metabolism is involved in the detrimental effect of microglial CARD19 deficiency on ischemic brain injury.

Whether microglial apoptosis is harmful or beneficial in ischemic stroke remains controversial. Traditionally, apoptosis was considered a non-inflammatory mode of cell death, characterized by the prevention of intracellular content leakage due to the preservation of membrane integrity (Tang et al., 2019). However, increasing evidence suggests that apoptosis results in the release of nuclear materials to the cell surface and into the extracellular environment (Radic et al., 2004; Jiang et al., 2007; Elliott et al., 2009; Montico et al., 2018; Murao et al., 2021). During apoptosis, microglia may release DAMPs (e.g., ATP and HMGB1) that promote neuroinflammation and neuronal damage (Gao et al., 2024). Therefore, microglia are now considered to be deleterious during the acute phase of ischemic stroke, and treatments that suppress microglia activation have been intensively explored (Qin et al., 2019; Tang et al., 2020a). However, selective elimination of microglia increases brain injury in ischemic stroke, which is reversed by microglial repopulation (Szalay et al., 2016; Marino Lee et al., 2021). These results emphasize the protective effect of microglia survival against ischemic brain injury. In this study, we found that CARD19 deficiency promoted microglial apoptosis *in vivo* and *in vitro*. Future studies could explore the influence of microglial apoptosis during the acute phase of ischemic stroke.

In addition, cerebral ischemic injury involves multiple signaling pathways, including those related to stem cell repair mechanisms. Promoting neurogenesis and the proliferation of endogenous neural stem cells/progenitor cells are considered promising strategies for encouraging neurological recovery following stroke. Recent studies have found that artesunate enhances functional recovery in mice after MCAO by promoting neurogenesis and proliferation of neural stem/progenitor cells via the FOXO3a/p27Kip1 axis, suggesting potential therapeutic options for ischemic stroke (Zhang et al., 2020, 2022). Whether microglial CARD19 is involved in neurogenesis after ischemic stroke remains unknown.

Mechanistically, we confirmed that CARD19 interacts with MIB components such as MIC19, SAMM50, and MIC60 in microglia. Mitochondrial cristae and cristae junctions are critical to mitochondrial morphology, structure, and function (Frey and Mannella, 2000; Cogliati et al., 2013). Many mitochondrial functions require strict regulation of mitochondrial cristae morphology, which is organized by MICOS (Eramo et al., 2020). MICOS is a multi-subunit protein complex that stabilizes the structure of the inner mitochondrial membrane and promotes mitochondrial homeostasis (Zerbes et al., 2012). The MICOS complex is formed by the assembly of two subcomplexes: the MIC60 subcomplex (composed of MIC60-MIC19-MIC25) and the MIC10 subcomplex (composed of MIC10-MIC26-MIC27). In human cell lines, the MIC60-MIC19-MIC25 subcomplex binds to the outer mitochondrial membrane proteins SAMM50 and metaxins 1/2 though MIC19, establishing the part of the MIB that spans the intermembrane space (Tang et al., 2020b). In recent years, defects in MIB assembly and activity have been found to be associated with a variety of neurological diseases, such as amyotrophic lateral sclerosis and Parkinson’s disease (Zeharia et al., 2016; Zhou et al., 2019; Li et al., 2020; Benincá et al., 2021). The loss of MIB components causes mitochondrial dysfunction and structural abnormalities, as indicated by increased mtDNA release, an enhanced STING-dependent IFN-I response, and increased mitochondrial ROS production (He et al., 2022; Li et al., 2024a). Our results suggest that the interaction between CARD19 and the MIB complex likely underlies the exacerbation of mitochondrial damage in response to microglial CARD19 deficiency. However, the role of CARD19 promotion of MIB component binding in ischemic stroke was not explored in this study, and requires further investigation.

Our findings suggested that MIC19 binding to SAMM50 was disrupted by OGD/R in microglia. CARD19 overexpression may compensate for the strength of the MIC19 interaction with SAMM50 to maintain mitochondrial cristae structure. The interaction of mitochondrial membrane proteins in the MIB regulates cristae morphology by controlling the formation and size of cristae junctions (Wollweber et al., 2017). The SAMM50-MIC19-MIC60 axis connects the sorting and assembly machinery and MICOS complexes to assemble the MIB super-complex, which mediates contact between the outer and inner mitochondrial membranes (Tang et al., 2020b). MIB depletion leads to mitochondrial cristae disorganization (He et al., 2022). Disruption of the SAMM50-MIC19-MIC60 axis also leads to mitochondrial morphological abnormalities, loss of mitochondrial cristae connections, abnormal cristae distribution, and reduced ATP production. In a rat model of intracerebral hemorrhage, binding between MIC19 and SAMM50 was disrupted after intracerebral hemorrhage. MIC19 overexpression alleviates neuronal apoptosis, mitochondrial structure and function abnormalities, oxidative stress in mitochondria, and neurobehavioral defects after intracerebral hemorrhage (Yang et al., 2023). Loss of SAMM50 in hepatocytes induces cardiolipin-dependent mitochondrial membrane remodeling, which triggers mtDNA release and liver injury (Chen et al., 2022). MIC19 deletion leads to mitochondrial ultrastructural changes, including mitochondrial cristae abnormalities and loss of cristae connections, which impairs ATP production (Darshi et al., 2011; Sohn et al., 2023). Combined with the strengthened interaction of SAMM50 and MIC19 in microglia overexpressing CARD19, it can be concluded that CARD19 facilitates assembly of MIB components, thereby preserving mitochondrial cristae structure.

This study has some limitations that should be noted. Firstly, this study focused solely on male mice during the acute phase of stroke (1 and 3 days after MCAO). To enhance clinical relevance, future research should consider including female mice and additional significant time points (e.g., 7 or 14 days post-stroke). In addition, the cortical stereotaxic injection of AAVs performed in this study did not knock down CARD19 expression in all microglia. Future studies could utilize Cre-LoxP mice to knock out CARD19 expression in all microglia. Finally, as CARD19 is also expressed in other brain cells, such as neurons, we plan to investigate the role of CARD19 in ischemic stroke in these cell types in the future.

Taken together, our findings suggest that microglial CARD19 ameliorates ischemic brain injury by stabilizing mitochondrial cristae. A better understanding of the microglial CARD19 function and mechanisms after ischemic brain injury may provide valuable insights into novel therapeutic targets for the treatment of ischemic stroke.

## Additional files:

***Additional Figure 1:*** *AAVs transfected microglia and specifically knocked down CARD19 in vivo.*

Additional Figure 1 AAVs transfected microglia and specifically knocked down CARD19 *in vivo*.
(A-C) Representative immunofluorescence images of AAV (mCherry) localization within neurons (NeuN; A), astrocytes (GFAP, B), and endotheliocytes (CD31, C). Scale bars: 50 μm. (D) Western blot analysis of CARD19 expression in the cortical tissue of mice from the AAV-control and AAV-shCARD19 groups 3 days after MCAO. *n* = 3/group. (E) Quantitative analysis of the data shown in Figure 4D. *n* = 3/group. (F) Quantitative analysis of the data shown in D. n=3/group. All experiments were repeated at least three times, with each using a separate brain sample. Data are presented as mean ± SD. In E, F, two-tailed unpaired Student's *t*-test was used. ^*^P < 0.05. AAV: adeno-associated virus; CARD19: caspase activation and recruitment domain 19; DAPI: 4',6-diamidino-2-phenylindole; GFAP: glial fibrillary acidic protein; NeuN: neuron-specific nuclear protein.

***Additional Figure 2:*** *Quantification of CARD19 and heat shock protein 60 co-localization in microglia.*

Additional Figure 2 Quantification of CARD19 and heat shock protein 60 co-localization in microglia.
(A) Scatterplot of the co-localization data shown in Figure 6B. (B) 3D reconstruction of Figure 6B. Scale bar: 4 μm. CARD19: Caspase activation and recruitment domain 19.

***Additional Table 1:*** *Primers used for quantitative polymerase chain reaction analysis.*

## Data Availability

*All relevant data are within the paper and its Additional files*.
