# Supplementary figures and images for: Microglial CARD19 ameliorates post-stroke neuroinflammation by stabilizing mitochondrial cristae
Source: Neural Regen Res. 2025 Mar 25;21(7):2975–85. doi: 10.4103/NRR.NRR-D-24-00923 (PMC13378934; doi:10.4103/NRR.NRR-D-24-00923)

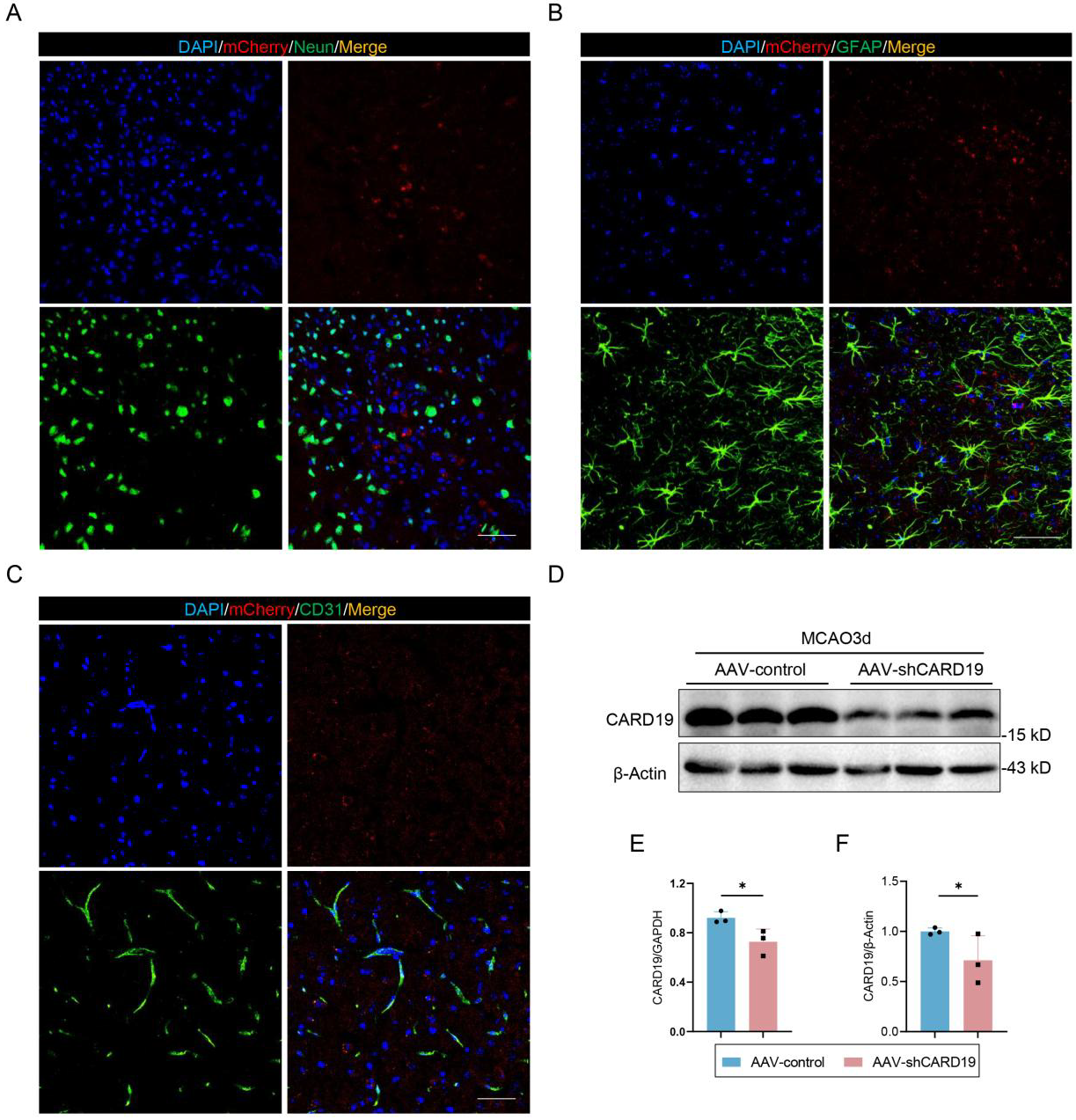

Supplement: Supplementary file 1 [file NRR-21-2975_Suppl1.tif]

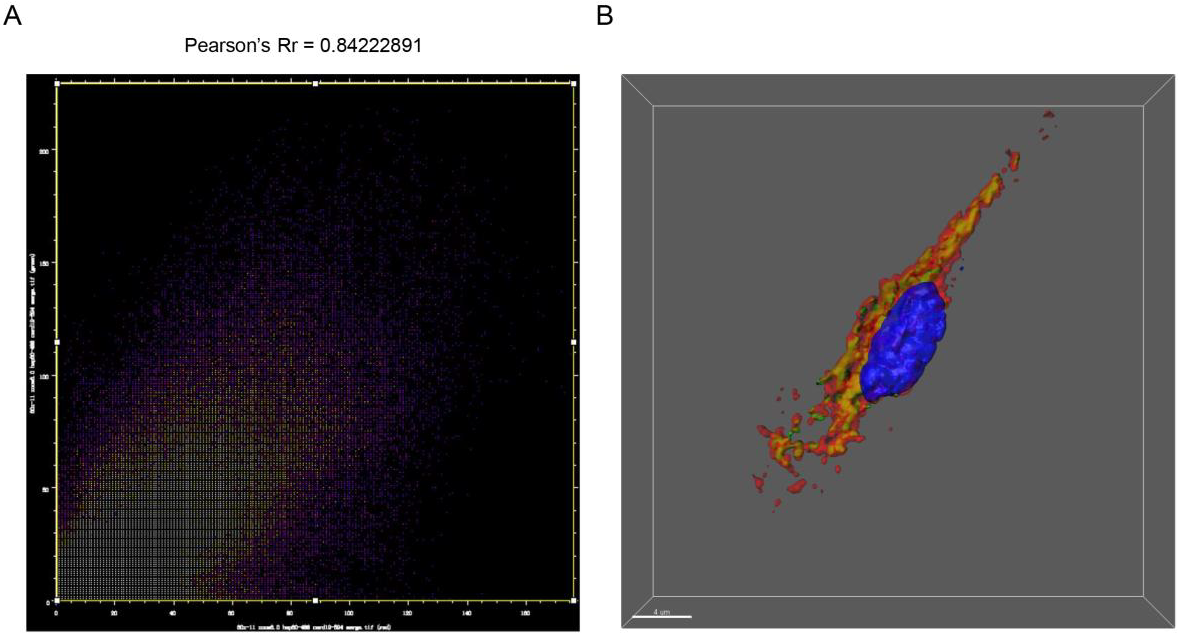

Supplement: Supplementary file 2 [file NRR-21-2975_Suppl2.tif]
